# A Practical Treatise upon Diseases of the Ear

**Published:** 1885-06

**Authors:** 


**A Practical Treatise upon Diseases of the Ear.** Including a Sketch of Aural Anatomy and Physiology. By D. B. ST. JOHN ROOSA, M D., LL. D , Professor of Diseases of the Eye and Ear in the New York Post Graduate School, etc. Sixth edition. Revised and enlarged. New York: Wm. Wood & Co. 1885.

Of a book, which, in eleven years, has reached a sixth edition, it is superfluous to say that it is a good one. The author, however, informs us in his preface that many pages have been added, and hardly a page escaped alteration. The larger part of the work is essentially a digest of its distinguished author's experience, and has thus increased in value with every successive edition. At the same time, he does not ignore the experience of other physicians, both of this country and Europe, and the present volume will fully sustain the high position so universally accorded to the work as without a rival in its special field.

